# Single-Stage Revision for Treatment of Prosthetic Joint Infection in Total Hip Arthroplasty

**DOI:** 10.1007/s43465-025-01405-6

**Published:** 2025-05-21

**Authors:** H. Humphries, W. Wignadasan, A. Fontalis, A. Alsheddi, M. Shaeir, F. S. Haddad

**Affiliations:** 1 Department of Trauma and Orthopaedic Surgery, University College London Hospitals, London, UK; 2 NHS Foundation Trust, London, UK; 3 Division of Surgery and Interventional Science, University College London, London, UK

**Keywords:** Arthroplasty, Infection, Revision, Single stage

## Abstract

Total hip arthroplasty (THA) is a highly successful procedure for managing hip osteoarthritis, with increasing numbers performed yearly. Prosthetic joint infection (PJI) is a devastating complication which remains one of the leading causes of implant failure and is increasing in prevalence, resulting in significant morbidity, mortality, and economic burden. Traditional management of PJI has relied on two-stage revision, but recent evidence suggests that single-stage revision may be an effective alternative for select patients. This review looks at the pathogenesis, diagnosis and classification of PJI in THA, and examines the current literature on single-stage revision for PJI in THA, including its indications, surgical technique, and clinical outcomes. Indication of the procedure is determined by the patient comorbidities and biology, and micro-organism characteristics. The surgical technique involves the removal of infected implants, thorough debridement, and reimplantation of a new prosthesis within the same procedure. Single-stage revision is associated with reduced hospital stays, lower costs, and improved patient satisfaction compared to two-stage revision. While successful eradication of infection depends on factors such as host immunity, pathogen virulence, and soft tissue viability, studies have demonstrated comparable or superior infection-free survival rates for single-stage revision. Recent advances in microbiological diagnostics, biofilm disruption techniques, and multidisciplinary perioperative management have further enhanced the efficacy of single-stage revision. Despite some contraindications, including highly resistant organisms and severe soft tissue compromise, high-volume centers have expanded their indications with promising results. As the demand for THA continues to rise, optimizing PJI treatment strategies is critical. This review highlights the growing role of single-stage revision as a viable and cost effective approach for managing PJI in carefully selected patients.

## Introduction

Total hip arthroplasty (THA) is a successful procedure that aims to improve pain and function in patients suffering from osteoarthritis of the hip [1]. With an increase in the aging population as well as demand for THA in younger, more functional demographics, it is estimated that there will be a 176% increase in THA from 2019 to 2040 [2]. Prosthetic joint infection (PJI), which is among the top three causes of THA failure, is a devastating complication that significantly increases morbidity, mortality, and healthcare costs [3–6]. PJI as a reason for reoperation is gradually increasing, with the Swedish arthroplasty registry finding it the most common indication for reoperation within 2 years of primary THA [7].

A worldwide meta-analysis of databases estimates the incidence of PJI following THA as 1.05%, but also found that databased-based studies underestimate the incidence of PJI [8]. Registry data has estimated the incidence of late PJI in THA to be 0.057% per prosthesis year [9, 10]. The increasing prevalence of comorbidities such as diabetes and obesity is expected to further increase the risk of PJI [10].

PJI poses a significant burden not only on patients, but also on the healthcare system, leading to extended hospital stays, potentially multiple surgeries, and a decline in patient quality of life [3, 10, 11]. The combined annual hospital costs associated with PJI of the knee and the hip were estimated to be $1.85 billion in the USA by 2030 [12]. In Europe, the mean direct hospital cost of procedures to treat THA PJI was $28,904 [13]. These costs often only take into account the hospital-associated costs and not the community healthcare costs and loss of patient productivity, so therefore likely do not reflect the true financial burden. Compared to aseptic cases, revisions for THA PJI have been shown to have increased cost, more readmissions, and a longer length of inpatient stay [14]. Furthermore, the morbidity and mortality associated with THA PJI are significant, with an up to 3.7 times increased risk of death within the first 2 years after diagnosis [15].

The management of PJIs post-THA involves the use of antibiotics and surgical intervention. Surgical procedures can comprise of a DAIR (debridement, antibiotics and implant retention) procedure with or without exchange of bearings, a single-stage revision exchange or a two-stage revision arthroplasty, or excisional arthroplasty [16]. Arthrodesis and amputation are considered last-resort treatment options [4]. PJI treatment is tailored to multiple factors, including whether the infection is acute or chronic, the virulence of the infecting organisms, host/patient factors, technicalities of the implant, and surgeon preference [17]. A two-stage revision exchange has been the standard for management of PJIs; however, a single-stage revision approach can serve as a feasible alternative for carefully selected patient groups [18]. Single-stage revision has shown comparable if not superior outcomes compared to two-stage revisions, fewer intraoperative complications, improved cost-effectiveness, reduced time in hospital, greater patient satisfaction, and reduced morbidity and mortality [17, 19–21].

A multidisciplinary team (MDT) is crucial in the management of PJI’s. Members of the team include a surgeon who specializes in revision arthroplasty, microbiologists, physiotherapists, dieticians and nursing staff [22]. An often underestimated factor is the stress that treating PJI’s has on the surgeon, and the MDT has been shown to ameliorate this burden [22]. Evidence has shown improved outcomes when PJI’s are treated in specialized arthroplasty centers with high-volume revision surgeons [23].

This review will look at the up-to-date evidence on single-stage revision for PJI in THA to provide a comprehensive overview of the topic, including the pathogenesis, diagnosis, and classification of PJI after THA, indications, contraindications, and advantages of single-stage revision, and the surgical technique involved.

### Pathogenesis, Classification, and Diagnosis of PJIs

#### Pathogenesis

PJIs predominantly occur in the first year following surgery due to inoculation of the pathogen onto the implant through direct or aerosolized contact at the time of operation [24]. They can also occur from contiguous spread from adjacent infected tissue, or through hematogenous dissemination from a peripheral infective source, commonly respiratory or genitourinary [24]. The common underlying mechanism in PJI is the formation of a biofilm. This is achieved by the expression of adhesion proteins by the infecting organism that adhere to the implant. The organisms then produce extra-cellular compounds which facilitate matrix formation and propagation of the organism colony [24]. Most important clinically is that the biofilm protects the bacteria from the action of antibiotics, making systemic antibiotic treatment of the microbes difficult. Orthopedic implant materials are susceptible to colonization by biofilm-forming bacteria, and include cobalt-chromium, titanium, polyethylene, polymethyl methacrylate (PMMA), and ceramics [25]. Commonly implicated bacteria in PJI are grouped as ESKAPE organisms (*Enterococcus faecium*, *Staphylococcus aureus*, *Klebsiella pneumoniae*, *Acinetobacter baumannii*, *Pseudomonas aeruginosa*, and Enterobacter species) [26]. The most common cause of PJI is *S. aureus*, followed by coagulase-negative staph, which overall are implicated in 50–60% of PJIs [24].

#### Diagnosis

Diagnosis of PJI in THA begins with a thorough history and examination. The classical signs of inflammation including pain, erythema, and swelling can help localize the infection to a specific joint. The presence of a sinus tract is classified by many as definitive evidence of a PJI [27, 28]. Chronic infections may be difficult to distinguish from aseptic failure based on clinical history and examination alone, as signs of infection may be lacking [29].

CRP and ESR are cost-effective serum markers valuable for diagnosing PJI and monitoring the clinical response after initiating treatment [24]. When measured together, their sensitivity and specificity for diagnosing PJI were 84% and 47%, respectively [30, 31]. The use of serum white cell count (WCC) is also routinely used in the diagnosis and follow-up of PJI; however, it is rarely elevated in chronic PJI, and its increasing value is nonspecific [30].

Synovial samples acquired through an aseptic technique play an important role in diagnosing PJI [30]. Synovial markers tested for include WCC and polymorphonuclear cell (PMN) percentage, leukocyte esterase, alpha defensin, and CRP [30]. Furthermore, culturing of synovial samples for aerobic and anaerobic microbes is essential for identifying the offending pathogen and their antibiotic sensitivity [27].

Plain film imaging, particularly useful when compared to prior films, is the first line imaging modality for diagnosing PJI [29]. Findings suggestive of PJI on plain films include radiolucency at the implant–bone interface, osteolysis, periosteal reaction, and implant migration. However, most of these findings are also present in patients with aseptic loosening, yielding a low sensitivity and specificity [29]. Other useful imaging modalities include computer tomography (CT), magnetic resonance imaging (MRI), positron emission tomography (PET), and bone scintigraphy [24, 29].

Emerging technologies designed to disrupt biofilms are enhancing the sensitivity of tissue culture testing. Sonication, which uses sound waves to break up biofilms and release bacteria, increases the chances of obtaining a positive culture from periprosthetic tissue[32]. A large prospective trial demonstrated an 18% improvement in sensitivity for infection diagnosis with sonication [33]. In addition, a meta-analysis confirmed its effectiveness in enhancing sensitivity for diagnosing PJI [34]. However, despite the positive results shown by sonication, it is yet to be widely accepted into the diagnostic armamentarium.

#### Classification

Over the years, multiple groups have continued to update and refine the criteria for reliably diagnosing PJI [28, 35, 36]. In broad terms, PJIs are classified into acute or chronic. In 2004, Zimmerli et al. classified PJIs into three groups: early PJIs (within 3 months postoperatively), delayed (3–24 months), or late (more than 24 months) [37]. Early PJIs typically present with rapid-onset joint pain, swelling, wound purulence, and fever, whereas chronic infections are more often characterized by low-grade pain, discomfort, sinus tract formation, and radiographic changes [38]. However, there is considerable variability and crossover in these presentations. Acute infections are generally caused by more virulent organisms such as *Staphylococcus aureus*, whereas chronic infections are often less virulent organisms [38]. The classification of PJIs as either ‘acute’ or ‘chronic’ is still widely utilized today.

In 2021, the European Bone and Joint Infection Society (EBJIS) formulated a classification system for PJIs (Table 1) [28]. This combines work from multiple groups including the Musculoskeletal Infection Society (MSIS) and the Infectious Diseases Society of America (IDSA) [35, 36]. The classification system integrates clinical features, laboratory investigations, and imaging to categorize the likelihood of infection into three groups: ‘unlikely,’ ‘likely,’ and ‘confirmed.’ [28] It was the first criteria to incorporate sonication into the diagnosis of PJI. As new technologies emerge, definitions and criteria for PJI continue to evolve, aiming to establish a universally accepted diagnostic standard [32].

**Table 1:** EBJIS criteria for the diagnosis of a PJI

	Infection unlikely—(no positive findings)	Infection likely (two positive findings)	Infection confirmed (any positive findings)
Clinical and blood work-up			
Clinical features	Clear non-infective reason defined for implant failure (eg fracture, tumour, position	1. Radiological evidence of implant loosening within five years of implantation 2. Previous wound healing issues 3. History of recent fever or bacteraemia 4. Purulence around the prosthesis	Sinus tract that communicates to the joint or visualization of the prosthesis
C-reactive protein		> 10 mg/L	
Synovial fluid analysis			
Leucocyte count (cells/µL)	≤1500	≥ 1500	≥ 3000
PMN (%)	≤65%	≥ 65%	≥ 80%
Synovial fluid biomarkers			
Alpha-defensin			Positive immunoassay/lateral flow assay
Microbiology			
Aspiration fluid		Positive culture	
Intraoperative (fluid/tissue)	All cultures negative	Single positive culture	2 positive samples with the same microorganism
Sonication (CFU/mL)	≤ 65%	≥ 65%	≥ 80%
Histology			
High-power field (400x magnification)	Negative	≥5 neutrophils in a single HPF	≥ 5 neutrophils in ≥5 HPFs
			Presence of visible microorganisms
Others			
Nuclear imaging	Negative three-phase isotope bone scan	Positive WBC scintigraphy	

### Indications for Single-Stage THA Revision

The single-stage revision for PJI was first described by Carlsson et al. [39], and has slowly gained popularity, particularly over the last 2 decades. It has been shown to be associated with decreased healthcare costs and reduced morbidity and mortality [4, 11, 17]. Studies have shown comparable if not superior outcomes compared to the two-stage revisions with regards to infection-free success [11, 19, 40].

There is no universal approach to managing PJIs, as treatment must be tailored to patient-specific and microorganism-related factors. Indications for a one-stage exchange include a non-immunocompromised host, absence of systemic sepsis, minimal bone/soft tissue loss allowing primary wound closure and a known pathologic organism with known antibiotic sensitivities preoperatively[17, 21]. Other high-volume centers have published their own indications [41–44].

Host comorbidities as well as local factors play an important role in determining the treatment of choice for PJI [11]. The McPherson classification categorizes host classes into groups: A, B, and C [45]. Class A are patients with no comorbidities, class B patients have one or two major comorbidities, and class C has three or more comorbidities or are essentially immunocompromised [45]. Wolf et al. categorized their patients according to the McPherson grading system when comparing single- and two-stage revisions for PJI in THA. In patients classified as class B and C at 2-year follow-up, one-stage revision eradicated infection in only 33.3% of cases, whereas two-stage revision was successful in 94.4% [46]. Other authors have excluded immunocompromised patients from single-stage revision [17]. Bori et al. showed a 95% infection-free success rate at 1 year following a one-stage revision. They noted a lack of bony defects in these cases, speculating that this may have contributed to the success rate [47]. The role of body mass index (BMI) in the success of single-stage revision has not been clearly identified; however, some studies have suggested a higher BMI is associated with an increased risk of failure [48].The patients’ local and systemic biology, therefore, has a large impact on the success of single-stage revision.

Microbiological analysis is used to confirm a target pathogen to guide antibiotic treatment and has been considered a key factor in determining the success of single-stage revision [49]. It is suggested that single-stage revision should not be performed if the organism and sensitivities are not known pre-operatively as antibiotic treatment cannot be targeted [40]. However, studies have shown successful infection eradication in single-stage revisions even when performed with negative pre-operative cultures [47, 50, 51]. This suggests that lack of pre-operative microbiological data may be a relative contraindication rather than an absolute contraindication to single-stage revision, with the emphasis being on the peri-operative microbiological confirmation [11]. Highly virulent organisms such as methicillin resistant *Staphylococcus aureus* (MRSA), polymicrobial infections, and rare organisms have been shown to make the success of single-stage revision less achievable [40]. The presence of a sinus tract has been thought to be a contraindication to single stage; however, studies have shown success with single-stage revision even in its presence [11, 52].

The ENDO-Klinik, a specialized, high-volume revision arthroplasty center in Hamburg, has shown successful results when performing a one-stage revision exchange even when multiple relative contraindications are present [41, 53]. They have treated up to 85% of patients with PJI of the hip with single-stage revision approach [54]. Registry data must be interpreted carefully due to patient selection bias and differences in data coding between hospitals [55].

DAIR is an alternative procedure used for the treatment of PJI, usually employed in the acute infective period when the infection arises within 4 weeks following surgery [56]. When compared with exchange arthroplasty, DAIR has lower morbidity as well as reduced cost when successful [56]. Two-stage DAIR procedures have shown comparatively high success rates in regards to implant retention when compared with single-stage DAIR; however, the two-stage approach may provide extended periods before reinfection [56]. Repeated DAIR is usually in the setting of failed initial DAIR which have shown variable success rates, and hence should be considered with caution when contrasting with the decision to embark on exchange arthroplasty [56]. While DAIR is generally used for early post-operative infections, Liukkonen et al. showed an almost halved risk of reinfection when single-stage was used compared to DAIR in this patient group [57], indicating potentially broader indications of single-stage revision time frames.

### Surgical Technique

Single-stage revision THA entails removing the existing prosthesis, performing extensive debridement, and implanting a new prosthesis in a single surgical procedure. The procedure is divided into four main stages: (1) approach and preparation (2) debridement and explantation of the infected prosthesis, (3) irrigation with local antiseptics, and (4) insertion of the new prosthesis [43, 58].

Preparation begins with the setup and positioning of the patient on the operating table. The pelvis is held in pace with bolsters allowing free range of movement of the affected limb in all planes [49]. Any hair overlying the surgical site is removed with a shaver, followed by a superficial clean [42]. The skin is prepped and draped in sterile fashion while the operating equipment is setup. The posterolateral approach to the hip is favored for its extensive exposure of both the acetabulum and femur [49].

The debridement stage involves both mechanical and chemical debridement. The incision is made excising the previous scar and any sinus tract if present, and is carried out down to the hip joint capsule [4, 49, 59]. During the approach, a minimum of three deep and superficial tissue and fluid samples should be taken and sent for aerobic, anaerobic, and extended cultures [60]. A radical synovectomy is performed, along with removal of the implant and any remaining foreign material which may harbor infection including cement and cement restrictors [17, 59]. Specific revision kit may be required to successfully explant the components safely. Any remaining necrotic bone or tissue must also be removed.

Following debridement, the wound must be irrigated, using a systemic approach. We recommend a 12L wash using warm 0.9% saline using a low-pressure pulsatile lavage to remove gross contamination [42, 59]. Bathing of the surgical field with an antiseptic solution of povidone-iodine commonly follow normal saline lavage [42, 49, 61]. A randomized controlled trial (RCT) demonstrated that dilute betadine lavage was significantly more effective in reducing the risk of PJI compared with normal saline lavage in patients undergoing aseptic revision hip and knee arthroplasty [62]. Gauze soaked with povidone-iodine is then packed into the wound with the skin edges reapproximated temporarily, allowing time for the iodine to fully exert its antimicrobial effects. Once satisfied that the site is free of contamination, a sterile drape is placed over the wound and a new surgical setup is carried out to provide a new sterile environment for reimplantation of the prosthesis [49]. Contaminated instruments are removed, the surgeons re-scrub, the surgical site is prepped and draped again, and fresh instruments are opened [43, 49, 58]. Intravenous antibiotics may be readministered at this point [49].

Reimplantation begins by preparing the acetabulum and femur for the revision prosthesis. At our institution, the majority of revisions are with cementless implants, with cement only used in significant osteoporotic bone with increased risk of intraoperative fracture [58]. Excellent results have been achieved with cementless implants for septic revisions [4, 50, 51]. Autogenic or allogenic cancellous bone grafting can be used with 3.64% vancomycin per weight of the bone graft in the case of deficient bone stock [43]. Alternatively, Tantalum augments can be used to address the bone defects [59]. In osteoporotic bone, antibiotic-impregnated bone cement can be used for the stem with a cementless acetabular component and tantalum augments for cases with significant bone loss [58]. Antibiotics that are added to the cement should be bactericidal and effective against the pathogens identified pre-operatively [43, 49]. The antibiotic should be in powder form and should weigh less than 10% of the total cement powder to avoid interference with the cement mechanical properties [59]. It is important to consider the total dosage of local antibiotics to prevent systemic toxicity [49]. Local delivery of antibiotics at the site of infection has been shown to achieve higher tissue concentration doses [40, 44]. Following implantation of the new prosthesis, the surgical field is washed again thoroughly. As always, meticulous, water-tight closure of the wound is imperative.

In the post-operative phase, routine physio-led rehabilitation with early mobilization should begin on the ward with adequate nutrition and analgesia [59]. A patient-specific rehabilitation plan is important to tailor to the bony and soft tissue defects reconstructed during the operation [49]. Ongoing intravenous antibiotics usually for 2 weeks are recommended until the microbiology from the intraoperative samples is available, after which the antibiotics can be orally converted, tailored to the organism sensitivities [63]. The antibiotic management is carried out with guidance from the microbiology team. The minimum time frame for oral antibiotic treatment is 6 weeks post-operatively; however, the duration is adjusted on a case-by-case basis and may be extended if there is not deemed to be a suitable response. If a suitable oral antibiotic is not identified, broad-spectrum intravenous antibiotics can be continued in the community. Over this period, blood markers including C-reactive protein (CRP) and erythrocyte sedimentation rate (ESR) are monitored to assess response to infection [43]. If recurrence of infection is suspected, image guided aspiration may be necessary.

### Contraindications of Single-Stage Revision THA

Contraindications for single-stage revision surgery vary among authors and institutions. These include a culture-negative infection where bacterial sensitivities are unknown, inability to obtain adequate primary soft tissue coverage, infection involving the neurovascular bundles and other unresectable infected areas, systemic sepsis, and infection with a highly virulent organism [40, 41, 49]. Oussedik et al. described a criteria in their study that contradicts the utilization of a single-stage revision THA procedure and this is outlined in Table 2 [21].

**Table 2:** Contraindications to single-stage revision

Local factors	Host factors	Organism factors
• Significant compromise to soft tissue	• Immunosuppression	• Polymicrobial organisms
• Significant loss of bone precluding cement reconstruction	• Reinfection	• Multi-resistant organisms (MRSA)
• Peripheral vascular disease	• Systemic disease	• Atypical commensals
	• Concurrent sepsis	• Unusual resistance profiles
		• Unidentified infective organisms

### Advantages of Single-Stage Revision

There is an increasing body of literature showing comparable or superior outcomes of using a single-stage revision approach compared with the two-stage exchange, in terms of infection-free survival and functional outcomes [17, 20, 21, 64–66]. Advantages of a one-stage revision include reduced healthcare costs, decreased length of hospital stay, decreased morbidity associated with one rather than two operations, reduced duration on antibiotics, lower rates of complications such as dislocation, earlier return to work and activity, and higher patient satisfaction [4, 49, 54, 67, 68]. The two-stage approach has been shown to be 1.6 times more costly than single stage [69, 70]. An RCT comparing single-stage with two-stage revision demonstrated superior patient reported outcomes at 3 months, fewer intraoperative complications, and increased cost-effectiveness [19].

Single-stage revision has been shown to be effective at successful eradication of PJI. Oussedik et al. showed no recurrence of PJI of the hip in a cohort of 50 patients at a mean follow-up of 6.8 years, compared to a reinfection rate of 5.8% over the same time frame for two-stage revision patients [21]. RCT data showed no difference in infection rates between the two procedures at 18 months [19]. A literature review of over 1,200 single-stage revision showed an infection-free rate of 83% at 5 years [71]. An up-to-date systematic review of single- vs two-stage revision for PJI found no difference in reinfection rates [72]. When dealing with a PJI with a concurrent discharging sinus, single stage has shown to be effective, bringing into consideration its status as a relative contraindication [41, 49]. In a cohort of 57 patients undergoing single-stage cemented revision THA with a discharging sinus, they found an 86% success rate at an average of 7-year follow-up [73]. Furthermore, the ENDO-Klinik does not consider a sinus tract a contraindication to single-stage revision [49]. They also have shown encouraging results using a single-stage procedure in treating infection with polymicrobial and resistant organisms also [49]. Li et al. reported that among 19 patients undergoing single-stage revision for polymicrobial infection, only 2 experienced reinfection, yielding outcomes comparable to their single-organism cohort [74].

Functional outcomes appear to favor single-stage revisions. In a retrospective propensity score-matched cohort study, Tirumala et al. matched 46 patients undergoing single-stage revision to 92 patients undergoing two-stage revision. The single-stage cohort showed statistically superior scores than the two-stage revision cohort for every patient reported outcome measure (PROM) included in the study [68]. Oussedik et al. showed significantly improved Harris Hip scores at 5 years for single-stage revisions [21]. Higher quality of life scores was shown at a mean follow-up of 41.3 months in single-stage revision [64].

### Artificial Intelligence and Predictive Analytics

Emerging technologies such as artificial intelligence (AI) and predictive analytics are increasingly being explored to enhance decision-making and outcomes in single-stage revision for PJI following THA. AI-driven technology such as machine learning (ML) models can synthesize complex clinical data from large patient datasets to aid in patient selection, prognostication, and peri-operative planning. This may help identify patients most likely to benefit from a single-stage approach and enhance the efficacy of treatment strategies [75, 76]. In studies on prediction of high risk patients for PJI, ML models frequently outperform traditional risk calculators [76, 77]. In the diagnosis of PJI, ML algorithms have achieved high diagnostic accuracy which can significantly improve diagnostic efficiency [78]. In regards to peri-operative planning, AI tools facilitate optimized surgical strategies and implant selection, and AI-driven robotic surgery achieves more precise osteotomy, overall improving treatment outcomes [76]. However, in AI research in PJI, the studies are often based on limited cohort sizes with mainly retrospective studies and lack external validation, potentially inducing bias and leading to reduced generalizability; therefore, further research with judicious clinical oversight is required. While still in early phases of clinical integration, these technologies are likely to shift the paradigm toward more personalized, data-driven revision strategies when treating PJI.

## Conclusion

Periprosthetic joint infection (PJI) is a devastating complication of total hip arthroplasty (THA). With the rising number of THA procedures performed annually, PJI will remain a significant challenge, placing a continued burden on both healthcare systems and patients. Compared with the two-stage exchange, the single-stage exchange has the clear benefits of reduced hospital stay, better cost-effectiveness, and faster return to function for patients. While the two-stage revision approach has been considered the gold standard for treating PJI, single-stage revision is a viable alternative for carefully selected patients.
